# Medical appropriateness of adult calls to Danish out-of-hours primary care: a questionnaire-based survey

**DOI:** 10.1186/s12875-017-0617-1

**Published:** 2017-03-14

**Authors:** Karen Busk Nørøxe, Linda Huibers, Grete Moth, Peter Vedsted

**Affiliations:** Department of Public Health, Research Unit for General Practice, Aarhus University, Bartholins Alle 2, 8000 Aarhus C, Denmark

**Keywords:** Denmark, After-hours care, Primary health care, Health care seeking behaviors, Delivery of health care, Health care utilisation

## Abstract

**Background:**

Optimal utilisation of the out-of-hours primary care (OOH-PC) services remains a concern in public health policy. We need more knowledge on potentially avoidable contacts. This study examines the frequency of medically assessed inappropriate OOH-PC calls from adults, explores factors associated with such assessment, and examines the relation to patient-assessed severity of health problem and fulfilment of expectations.

**Methods:**

We performed secondary analyses of data from a large cross-sectional survey on contacts to Danish OOH-PC. As access to Danish OOH-PC is provided through telephone triage delivered by a general practitioner (GP), we included only telephone contacts. A contact was characterised as medically inappropriate when the triage GP assessed that the request from a medical perspective should have been directed to daytime primary care. Appropriateness was examined in relation to patient characteristics, reason for encounter, time of contact, and whether the contact was triaged to a face-to-face consultation, and in relation to patient-assessed severity of the health problem and fulfilment of expectations. Associations were estimated with odds ratios (ORs) using multivariate analysis.

**Results:**

Of all contacts, 23.7% were assessed as medically inappropriate. Such assessment was associated with: younger age, longer symptom duration, exacerbation of chronic condition, and contact only few hours away from own GP’s office hours. Of medically inappropriate contacts, 31.3% were from patients aged 18–30 years, 41.5% concerned symptoms of > 24 h, 19.4% concerned exacerbation of chronic condition, and 21.3% were calls < 3 h away from own GP’s regular office hours. Medicine request was the most frequent reason for an inappropriate contact (14.3% of medically inappropriate contacts). In 53.4% of contacts assessed as inappropriate, the health problem was considered as severe by patients and medical assessed inappropriateness was significantly associated with unfulfilled patient expectations.

**Conclusions:**

One in four OOH-PC calls was considered medically inappropriate. Future efforts to reduce suboptimal use of OOH-PC should focus on the types of contacts with the highest optimisation potential, e.g., medication requests, long-lasting symptoms, and exacerbations. Such interventions should aim at bridging the gap between the GP’s medical assessment and the patient’s expectations to appropriate OOH-PC use.

## Background

Patient contacts to out-of-hours primary care (OOH-PC) are sometimes perceived as medically inappropriate by health care professionals [1–3]. A few contacts should have been directed to an emergency department (ED), some are considered manageable by self-care, and others should have been presented within the regular office hours in primary care [4, 5]. As the demands for OOH-PC are high, a major health policy concern is to reduce the suboptimal use of these services [6]. It is frequently debated how to lower the number of inappropriate contacts without endangering the health and safety of the patient. However, ‘inappropriate’ use of acute health-care services is a complex issue, and the concept is poorly defined [7, 8]. In this study, we focus on contacts that according to a medical assessment made by the triage GP should have been directed to primary care within the regular office hours.

The organization of day-time and out-of-hours primary care affects the patients’ use of OOH-PC. Lower availability and accessibility of primary health care during daytime has been shown to cause increased use of acute care; this also includes OOH-PC services [9–12]. The direct access in Danish OOH-PC to a general practitioner (GP) who promptly performs telephone triage may stimulate patients’ use of OOH-PC as this direct access to medical care may be preferred over their own GP. In addition to the organisation of health care, the help-seeking behaviour among the patients depends on many other determinants, such as sociodemographic and cultural factors, previous experience, attitude to health-service use, and perception of symptoms [8, 13–16]. Patients often perceive their symptoms as more severe and urgent than health-care professionals [3, 8, 17–19].

Discrepancies between patients’ and GPs’ assessment of severity of symptoms and appropriateness of requests for OOH-PC may limit the potential of reducing the OOH-PC contacts that are considered inappropriate by GPs. The different views may also lead to poor fulfilment of the patients’ expectations for care. Development of interventions that may target medically inappropriate (and potentially avoidable) use of OOH-PC requires a deep understanding of the requests for OOH-PC services that are put forward by patients [6]. In particular, we need to study the medical assessment of inappropriateness in relation to the patient-assessed severity of the problem presented. However, to our knowledge, the existing information about medical appropriateness of patient use of acute care is sparse and mainly concerns ED [3, 9, 20].

The aims of this study were (1) to describe the frequency and the characteristics of “medically inappropriate” OOH-PC calls performed by adult patients, (2) to identify factors associated with contacts assessed by GPs as medically inappropriate, and (3) to examine the patient-assessed problem severity and the fulfilment of expectations in relation to the GP-assessed medical inappropriateness of the contact.

## Methods

### Study design and setting

The study was based on a large cross-sectional survey on patient contacts to OOH-PC in the Central Denmark Region (CDR) over a period of 12 months in 2010–2011 (LV-KOS 2011) [21]. Data was collected using questionnaires completed by GPs and patients and from the OOH electronic registration system.

Denmark is divided into five regions, and each is responsible for organising its own OOH-PC. In four of the regions, OOH-PC is organized by GPs in large-scale cooperatives; this also includes the CDR with 1.2 million inhabitants. Patients access OOH-PC by telephone, and GPs answer all patient calls and perform triage. Only fully licensed GPs perform triage. The triage GPs can manage the patient’s health problem by telephone (telephone consultation) or refer to a subsequent face-to-face contact (clinic consultation or home visit) or to hospital (ED or admission). The OOH-PC service is tax-paid and free for patients. The GPs are paid a fee-for-service. The service is available from 4 pm to 8 am on weekdays plus weekends and national holidays; it is intended to deal with requests for urgent medical assistance that cannot wait until regular office hours [22–24].

### Data collection

#### GP pop-up questionnaire

GPs were electronically invited to participate in the study when logging onto the OOH computer system at the start of a shift. Of all GPs doing telephone shifts, one GP per 8-h shift could participate. A short pop-up questionnaire integrated into the existing electronic patient administration system appeared after every 10th telephone contact. The registered contacts have been shown to be highly representative for all OOH-PC contacts in the CDR as patients included in the study varied less than three per cent compared with all patients in the study period regarding the distribution of age and gender [21].

The GP questionnaire included the question: ‘Should the patient from a medical perspective have contacted his/her own GP during daytime?’ The response categories were: ‘Yes’, ‘No’ and ‘Don’t know’. We defined ‘Yes’ as a contact assessed by GPs as medically inappropriate for OOH-PC. The GPs also stated triage outcome (telephone consultation or subsequent face-to-face contact in OOH-PC or at hospital), symptom duration, and estimated severity of the health problem by answering additional questions (as seen in Table 1). Finally, the GPs stated whether the contact concerned exacerbation of known chronic disease, new event (including symptoms of longer duration), or other reason (could be specified in text), and the specific reason(s) for encounter (RFE) were given. The stated RFEs were subsequently coded according to the International Classification of Primary Care (ICPC) [25]. RFEs coded with process code’- 50’ (medication - prescription/request/renewal/injection) were combined across organ systems into one category named ‘Request for medication’.

**Table 1:** Characteristics of all OOH-PC telephone contacts and frequency of contacts assessed as medically inappropriate

	All OOH-PC contacts	Contacts assessed by triage GP as medically inappropriate	
	N (%)	n (%)	n/100 contacts (95% CI)
All contacts	5333 (100)	1266 (100)	23.7 (22.6–24.9)
Level of care			
Telephone consultation only	2884 (54.1)	993 (78.4)	34.4 (32.7–36.2)
Triaged to face-to-face contact in OOH-PC	2188 (41.0)	258 (20.4)	11.8 (10.5–13.2)
Triaged to ED or hospital admission	261 (4.9)	15 (1.2)	5.7 (3.3–9.3)
Patient characteristics			
Sex			
Male	2336 (43.8)	539 (42.6)	23.1 (21.4–24.8)
Female	2997 (56.2)	727 (57.4)	24.3 (22.7–25.8)
Age groups (years)			
18–30	1410 (26.4)	396 (31.3)	28.1 (25.8–30.5)
31–40	1010 (18.9)	251 (19.8)	24.9 (22.2–27.6)
41–60	1368 (25.7)	350 (27.6)	25.6 (23.3–28.0)
61–75	786 (14.7)	168 (13.3)	21.4 (18.6–24.4)
> 75	759 (14.2)	101 (8.0)	13.3 (11.0–15.9)
Symptom characteristics (medical perspective)			
Top 10 RFE for medically assessed inappropriate contacts^a^			
1. Request for medication (-50)^b^	334 (6.3)	181 (14.3)	54.2 (48.7–59.6)
2. Fever (A03)	440 (8.3)	75 (5.9)	17.0 (13.6–20.9)
3. Cough (R05)	257 (4.8)	71 (5.6)	27.6 (22.3–33.5)
4. Abdominal pain/cramps general (D01)	302 (5.7)	58 (4.6)	19.2 (14.9–24.1)
5. Throat symptom/complaints (R21)	193 (3.6)	47 (3.7)	24.4 (18.5–31.0)
6. Headache (N01)	189 (3.5)	45 (3.6)	23.8 (17.9–30.5)
7. Back symptom/complaint (L02)	137 (2.6)	37 (2.9)	27.0 (19.8–35.2)
8. Vertigo/dizziness (N17)	145 (2.7)	35 (2.8)	24.1 (17.4–31.9)
9. Nausea (D09)	155 (2.9)	33 (2.6)	21.3 (15.1–28.6)
10. Ear pain (H01)	100 (1.9)	33 (2.6)	33.0 (23.9–43.1)
Other RFE(s) (includes 276 different RFEs)	3532 (66.2)	754 (59.6)	21.3 (20.0–22.7)
Reason for contact			
New episode (including symptoms of long duration)	4120 (77.3)	860 (67.9)	20.9 (19.6–22.1)
Exacerbation of chronic disease	868 (16.3)	246 (19.4)	28.3 (25.4–31.5)
Other	345 (6.5)	160 (12.6)	46.4 (41.0–51.8)
Duration of symptoms			
< 5 h	1712 (32.1)	215 (17.0)	12.6 (11.0–14.2)
5–12 h	1158 (21.7)	171 (13.5)	14.8 (12.8–16.9)
12–24 h	782 (14.7)	170 (13.4)	19.3 (16.7–22.0)
> 24 h	1251 (23.5)	525 (41.5)	42.0 (39.2–44.8)
Don’t know	223 (4.2)	75 (5.9)	33.6 (27.5–40.2)
Not relevant	207 (3.9)	110 (8.7)	53.1 (46.1–60.1)
GP-assessed severity			
Potentially severe	1646 (30.9)	113 (8.9)	6.9 (5.7–8.2)
Not severe, but the patient is ill	2777 (52.1)	747 (59.0)	26.9 (25.3–28.5)
Patient is not ill	635 (11.9)	331 (26.1)	52.1 (48.2–56.1)
Don’t know	275 (5.2)	75 (5.9)	27.3 (22.1–32.9)
Time of contact (health service perspective)			
Weekday vs. weekend/public holiday			
Weekday	2829 (53.0)	829 (65.5)	29.3 (27.6–31.0)
Weekend/public holiday	2504 (47.0)	437 (34.5)	17.5 (15.0–19.0)
Time in relation to own GP’s regular office hours			
< 3 h after office hours	720 (13.5)	269 (21.3)	37.4 (33.8–41.0)
≥ 3 h after/before office hours	4193 (78.6)	852 (67.3)	20.3 (19.1–21.6)
< 3 h before office hours	420 (7.9)	145 (11.5)	34.5 (30.0–39.3)

^a^More than one RFE could be registered

^b^91.8% of contacts had only this RFE; the remaining 8.2% also had other concomitant RFE(s)

#### Patient questionnaire

A questionnaire was sent to the patient for each registered contact. The questions covered aspects of the contact to OOH-PC and the patient’s general health status. No questionnaire was sent to patients with publicly recorded protection against research participation, unknown postal address, ethically sensitive health problems (e.g., attempted suicide or terminal illness), earlier participation in the study or death registration. Patients received the questionnaire within 1 week after the OOH-PC contact and a reminder with a new questionnaire after 2 weeks if the first questionnaire had not been completed and returned.

From the patient questionnaire we included two items exploring the patient-assessed severity of the health problem and fulfilment of expectations. The patient-assessed severity of the problem at the time of contacting OOH-PC was recoded into two categories: ‘Severe’ (‘Severe and life threatening’ and’Severe, but not life threatening’) and ‘Not severe’ (‘Not severe, but I found it necessary to talk to a physician’ and ‘I was not ill, but had some questions’). Fulfilment of patient expectations was recoded into three categories: ‘A lot/Completely’ and ‘A little/Some’ and ‘Not at all’.

#### OOH electronic registration system

Information on time and date of contact and patient age and gender was collected from the OOH electronic registration system. Time of contact was recoded into a variable for day (‘Weekday’ and ‘Weekend/public holiday’) and a variable for time before/after the office hours of own GP (‘<3 h after office hours’, ‘≥ 3 h after or before office hours’, and ‘<3 h before office hours’). Patient age was categorized into five age groups: 18–30, 31–40, 41–60, 61–75, and > 75 years.

### Analysis

**Fig. 1:**
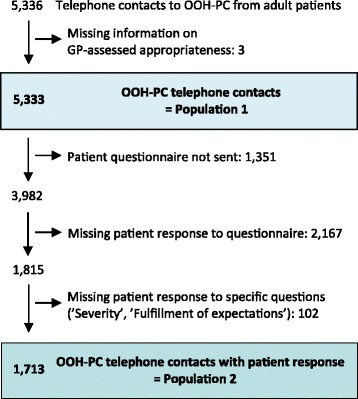
Flowchart presenting the study population

We included all registered telephone contacts from adults (age ≥ 18 years) (Fig. 1). Three contacts with missing information on appropriateness were excluded; this resulted in a study population of 5,333 OOH-PC patient calls (Study population 1). A total of 1,733 patients responded to the questionnaire, including the questions on problem severity and fulfilment of expectations (Study population 2). We also performed a non-response analysis.

Descriptive statistics were made for Study population 1 on the characteristics of the contacts assessed as medically inappropriate. The rate of contacts assessed as medically inappropriate was defined as the number of such contacts per 100 OOH-PC contacts. This rate was stratified for subgroups and calculated with 95% confidence interval (CI).

The association between contacts being assessed as medically inappropriate and contact characteristics was assessed using logistic regression. Included independent variables were patient characteristics (gender, age group), presented health problem (symptom duration, chronic condition), and time of contact (day, time in relation to regular office hours). Associations were adjusted for all mentioned variables, except for time in relation to office hours as this correlated with time of week.

Descriptive statistics were performed for patient-assessed severity and fulfilment of expectations (Study population 2). The association between medically assessed inappropriateness and both patient-assessed problem severity and fulfilment of expectations was analysed using logistic regression while adjusting for patient age and gender. The analyses of the associations with fulfilment of expectations were additionally adjusted for patient-assessed severity and triage outcome (telephone consultation or subsequent face-to-face contact in OOH-PC or at hospital) as these factors may be associated with patient evaluations of OOH-PC [26]. All associations were calculated as raw and adjusted odds ratios (OR) with 95% CI. *P*-values ≤ 0.05 were considered statistically significant. Analyses were performed in STATA, version 12.

## Results

### Contacts assessed by triage GPs as medically inappropriate

According to the triage GPs, 23.7% of contacts should have been directed to daytime primary care (Table 1). In 68.3% of cases, the contact should not have been directed to primary care, and the GP was unsure (‘Don’t know’) in 8% of cases (not shown in table). Of all OOH-PC calls, 45.9% were triaged to a subsequent face-to-face contact in OOH-PC or at hospital, whereas this was the case in only 21.6% of the contacts assessed as medically inappropriate.

Patients aged 18–30 years accounted for 31.3% of the medically inappropriate contacts, and 28.1% (95% CI: 25.8–30.5%) of all contacts from this age group were assessed as inappropriate. For patients aged 75 years or older, only 13.3% (95% CI: 11.0–15.9%) of contacts were assessed as inappropriate. In total, 41.5% of the inappropriate contacts concerned symptoms lasting > 24 h. In 26.1% of the medically inappropriate contacts, the GP assessed that the patient was not ill. In total, 286 different RFEs were registered for contacts assessed as inappropriate. Contacts concerning a medication request were assessed as inappropriate in 54.2% of cases (95% CI: 48.7–59.6%) and accounted for 14.3% of all inappropriate contacts.

### Factors related to GP assessed medical inappropriateness

As seen in Table 2, the ORs of assessed medical inappropriateness decreased with increasing patient age (adj. OR = 0.65 (95% CI: 0.51–0.82) for 61–75 years and adj. OR = 0.38 (95% CI: 0.29–0.50) for > 75 years). We found increasing ORs for longer symptom duration (adj. OR = 2.42 (95% CI: 1.92–3.05) for symptom duration of 12–24 h and adj. OR = 6.10 (95% CI: 5.02–7.40) for symptom duration of > 24 h) and for contacts concerning a chronic condition rather than a new episode (adj. OR = 1.29 (95% CI: 1.06–1.56)). Contacts during weekends and holidays were less likely to be assessed as inappropriate than contacts during weekdays (adj. OR = 0.36 (95% CI: 0.31–0.42)). Contacts taken < 3 h before or after own GP’s regular office hours were more often assessed as inappropriate than contacts taken ≥ 3 h away from daytime office hours (< 3 h after office hours: adj. OR = 1.63 (95% CI: 1.31–2.02) and < 3 h before office hours: adj. OR = 1.87 (95% CI: 1.44–2.42)).

**Table 2:** Associations between contact characteristics and medical inappropriateness (*N* = 4903^a^)

	Contacts assessed by triage GP as medically inappropriate			
	OR (95% CI)	P	Adj. OR (95% CI)^b^	P_Adj._
Patient characteristics				
Sex				
Male	1		1	
Female	1.07 (0.94–1.21)	0.313	1.09 (0.94–1.26)	0.275
Age (years)				
18–30	1		1	
31–40	0.85 (0.70–1.02)	0.076	0.81 (0.65–0.99)	0.046
41–60	0.88 (0.74–1.04)	0.137	0.86 (0.70–1.04)	0.116
61–75	0.70 (0.57–0.86)	0.001	0.65 (0.51–0.82)	< 0.001
> 75	0.39 (0.31–0.50)	< 0.001	0.38 (0.29–0.50)	< 0.001
Symptom characteristics				
Duration of symptoms				
< 5 h	1		1	
5–12 h	1.21 (0.97–1.50)	0.089	1.32 (1.05–1.64)	0.015
12–24 h	1.93 (1.55–2.41)	< 0.001	2.42 (1.92–3.05)	< 0.001
> 24 h	5.04 (4.20–6.04)	< 0.001	6.10 (5.02–7.40)	< 0.001
Reason for contact				
New episode (including symptoms of long duration)	1		1	
Exacerbation of chronic disease	1.50 (1.27–1.77)	< 0.001	1.29 (1.06–1.56)	0.010
Other	3.28 (2.62–4.10)	< 0.001	2.26 (1.60–3.21)	< 0.001
Time of contact				
Weekday vs. weekend/public holiday				
Weekday	1		1	
Weekend/public holiday	0.51 (0.48–0.58)	< 0.001	0.36 (0.31–0.42)	< 0.001
Time in relation to own GP’s regular office hours				
< 3 h after office hours	2.33 (1.98–2.77)	< 0.001	1.63 (1.31–2.02)	< 0.001
≥ 3 h after/before office hours	1		1	
< 3 h before office hours	2.07 (1.67–2.56)	< 0.001	1.87 (1.44–2.42)	< 0.001

^a^430 contacts in which the GP responded ‘Don’t know’ or ‘Not relevant’ to the question on symptom duration were omitted

^b^Adjusted for all presented characteristics of patients and symptoms and whether the contact was taken on weekday or during weekend/public holiday

### Patient-assessed severity and fulfilment of expectations

Patients assessed their health problem as severe in 53.4% of the medically inappropriate contacts and in 59.0% of all contacts (Table 3). Patient-assessed severity was not significantly associated with GP-assessed inappropriateness in the adjusted analysis (adj. OR = 0.80, 95% CI: 0.63–1.02). GP-assessed inappropriateness was significantly associated with unfulfilled patient expectations (adj. OR = 1.47 (95% CI: 1.09–2.00) for little/some fulfilment and adj. OR = 3.25 (95% CI: 2.13–4.85) for no fulfilment at all).

**Table 3:** Patient-assessed severity and fulfilment of expectations in relation to assessed medical inappropriateness of contact (Population 2, *N* = 1713)

	All OOH-PC contacts	Contacts assessed by triage GP as medically inappropriate				
	N (%)	n (%)	OR (95% CI)	p	Adj. OR (95% CI)^a^	p_Adj_.
Patient-assessed severity						
Severe	1011 (59.0)	187 (53.4)	0.77 (0.61–0.99)	0.038	0.80 (0.63–1.02)	0.077
Not severe	671 (39.2)	152 (43.4)	1		1	
Not ill, but had some questions	31 (1.8)	11 (3.1)	1.88 (0.88–4.00)	0.103	1.84 (0.86–3.94)	0.119
Fulfilment of expectations						
A lot/completely	1266 (73.9)	217 (62.0)	1		1	
A little/some	325 (19.0)	80 (22.9)	1.58 (1.18–2.11)	0.002	1.47 (1.09–2.00)	0.012
Not at all	122 (7.1)	53 (15.1)	3.71 (2.52–5.47)	< 0.001	3.25 (2.13–4.85)	< 0.001

^a^Analysis of patient-assessed severity adjusted for gender and age group. Analysis of fulfilment of expectations adjusted for gender, age group, patient-assessed severity, and whether the contact was managed by telephone consultation or triaged to face-to-face contact (OOH-PC or hospital)

The non-response analyses for this population showed a higher proportion of medical inappropriate contacts among patients who did not respond to the questionnaire than among responding patients (25.9% (95% CI: 24.1–27.8) vs. 20.4% (95% CI: 18.5–22.3)). The patient response rate was 45.6% (95% CI: 44.0–47.1) overall, but it was 33.7% (95% CI: 29.4–38.1) for patients assessed by the GP not to be ill.

## Discussion

### Main findings

About a quarter of OOH-PC telephone contacts should have been directed to daytime primary care according to the triage GPs’ medical assessments. Increasing symptom duration was strongly associated with GP-assessed medical inappropriateness, and as many as 40% of the inappropriate contacts were related to symptoms lasting > 24 h. Other characteristics associated with inappropriateness were younger patient age, exacerbation of chronic disease, contact during weekdays, and contacts taken < 3 h before/after own GP’s opening hours. The RFEs showed wide clinical variation, but medication requests were the most frequent RFE among the medically inappropriate contacts. More than half of the inappropriate contacts concerned symptoms that were considered severe by patients, and contacts assessed by GPs as medically inappropriate were significantly associated with unfulfilled patient expectations.

### Comparison with other studies

When comparing studies on inappropriate use of acute health care services it should be held in mind that there is no agreed definition of inappropriateness and that different measures are used [7, 8]. A review found that the prevalence of inappropriate ED use varied from 10 to 90% depending on the criterion used to define inappropriateness [9]. Research on frequency of inappropriate OOH-PC use is very sparse. To our best knowledge, we are the first to examine medical appropriateness of OOH-PC contacts by using triage GPs’ medical assessment of whether patients should have contacted their own GP. We found that about one of four OOH-PC contacts was assessed as medically inappropriate. A Norwegian study found that 28% of OOH-PC consultations concerned minor ailments that the patients could easily have handled themselves [4]. In contrast to the Norwegian study medically inappropriate contacts in our study may concern medically relevant requests that should already have been seen by the patients’ own GP.

The observed peak in the number of medically assessed inappropriate contacts during the first opening hours of OOH-PC and on weekdays compared with weekends may be explained by limited accessibility and availability of daytime primary care. This is supported by previous studies showing accessibility of daytime care to be associated with consumption of acute care, including OOH-PC services [9–12] Our finding may also reflect that patients are unable or unwilling to attend their own GP during office hours due to daytime commitments [14, 15, 27]. However, in a Dutch study of low-urgency OOH-PC contacts, only 1.5% of patients stated lack of time to go to the GP during the day as a reason for contacting OOH-PC [20]. The observed variation in medical accessed inappropriateness among different age groups may have to do with age-related differences in help-seeking behaviours and type and severity of health problems [28, 29]. Thus, the high rate of medically inappropriate contacts among young adult patients may partly be due to a high overall consumption of OOH-PC services along with a low likelihood of severe illness [30, 31]. The increasing likelihood of inappropriateness with younger age, which was observed in the present study, has also been identified in other studies concerning OOH-PC and ED [9, 20, 32].

As OOH-PC is intended to manage acute health problems, the observed association between long symptom duration and high rate of medically assessed inappropriateness was expected. Long symptom duration has previously been shown to be associated with inappropriateness [9, 20]. With regard to specific RFEs, we found much variation among both medically appropriate and inappropriate contacts. Other studies have also reported considerable variation in RFEs in OOH-PC [33, 34]. As contacts concerning medication request (incl. renewal of prescription) were assessed as inappropriate in more than half of cases and accounted for about 14% of all inappropriate contacts this RFE may be targeted in future effort to optimise OOH-PC utilisation. The gain from targeting specific RFEs other than medication request seems low as each RFE accounted for a low percentage of inappropriate contacts.

The disparity between patient-assessed severity and GP-assessed appropriateness reflects the known gap between patients and healthcare professionals concerning their understanding of urgency and severity of symptoms [3, 8, 17–19]. Also, the association between medically assessed inappropriateness and unfulfilled patient expectations could reflect the mismatch between the perception of appropriateness among the patients and the medical assessments provided by the GPs. The propensity among patients to use OOH care is not only influenced by medical factors but also by feelings (e.g., uncertainty, anxiety), ability to self-manage, practical concerns, and attitudes to and knowledge about health care [8, 13, 14, 16, 35].

Patient worrying and their need for reassurance are important motives for contacting the OOH-PC. Thus, the potential for reducing the number of medically inappropriate contacts to OOH-PC may be limited.

We assume that Danish citizens understand what the intended aim of OOH-PC is: health care for patients with acute health problems that cannot wait until office hours of the own GP. Information on how and when to use Danish OOH-PC including advice on appropriate alternatives is available online Yet, we do not know to what extent Danish citizens are aware of this intended aim. In a Dutch study 46.6% of patients calling OOH-PC for non-urgent health problems thought that the OOH-PC was intended for all health problems and it was suggested that society’s experiences with expanded opening hours of other services may have led to increased expectations of healthcare delivery [20].

### Strengths and limitations of the study

This study is based on a considerable number of randomly sampled and representative telephone contacts to OOH-PC, which provides our study results with high statistical precision. GP registrations were made immediately after the contact, which reduces the risk of recall bias. Furthermore, the included patients received the questionnaire shortly after their contact to OOH-PC.

The assessment as to whether a health request to OOH-PC is medically appropriate for the setting is not an objective assessment, and there may be some variation among GPs. However, the assessment was performed by fully licensed GPs who were asked to assess appropriateness from a medical perspective (as opposed to a more holistic perspective or a personal opinion). Thus, it can be considered a highly qualified assessment of medical appropriateness. As the triage GPs stated whether the patient should have contacted a GP within office hours, we could not distinguish between OOH contacts that should have been directed to the patient’s own GP already and those that could have been postponed. We chose this approach to minimize the triage GPs’ workload of responding to the pop-up questionnaire. We obtained a reasonably high response rate (45.6%) for the patient questionnaire, but some selection bias could still be present. The non-response analysis showed a higher proportion of medically inappropriate contacts among non-respondents than among respondents. Additionally, the response rate was lower for contacts in which the patient was assessed not to be ill by the GP. Therefore, the share of inappropriate contacts that were perceived as non-severe by the patients may have been underestimated. Using a more holistic phrasing of the question on appropriateness, including context factors, could possibly have increased the frequency of contacts assessed as appropriate; this may also have strengthened the association between GP-assessed appropriateness and patient-perceived severity. Yet, such phrasing may have reflected the individual GP’s personal opinion rather than a medical assessment of appropriateness.

### Recommendations and future research

Efforts made to adjust the help-seeking behaviours among patients and reduce the suboptimal use of the OOH-PC should focus on the types of contacts with the highest potential for optimization. It could be relevant to address both the general population and specific patient groups (e.g., young adult patients) to inform them about how and when to use the OOH-PC (e.g., ordering medication on time at their own general practice and contacting own GP during daytime if possible), including the overall aim of OOH-PC. However, more knowledge is needed about potentially avoidable calls to OOH-PC. Future studies should investigate patient perspectives on self-assessed urgency and motives for calling the OOH-PC rather than a GP within office hours in order to plan tailored interventions.

## Conclusion

Approximately one out of four calls to OOH-PC was assessed as medically inappropriate by the triage GP, which indicates a potential for optimising OOH-PC utilisation. Strategies should focus on contact types with the highest likelihood of medically assessed inappropriateness such as calls concerning medication requests or long-lasting symptoms and calls from young adult patients. However, the finding that patients frequently assessed the health problem severe and were more likely to report unfulfilled expectations in case of medically assessed inappropriateness emphasizes that ‘appropriate’ use of OOH-PC is a complex and multifaceted topic. Medical and patient perspectives are not necessarily consistent and strategies to optimise OOH-PC utilisation need to include both perspectives.

## Abbreviations

CDR: Central Denmark Region
CI: Confidence interval
ED: Emergency department
GP: General practitioner
ICPC: International Classification of Primary Care
LV-KOS 2011: Survey of reasons for encounter and disease patterns in Danish out-of-hours primary care conducted in 2011
OOH-PC: Out-of-hours primary care
OR: Odds ratio
RFE: Reason for encounter
